# The Versatility of Autologous Fat Transplantation in Correction of Facial Deformities: A Single-Center Experience

**DOI:** 10.1155/2015/703535

**Published:** 2015-03-03

**Authors:** Niels Hammer-Hansen, Javed Akram, Tine Engberg Damsgaard

**Affiliations:** Plastic Surgical Research Unit, Department of Plastic Surgery, Aarhus University Hospital, 8000 Aarhus, Denmark

## Abstract

Deformities in the craniofacial region are of great social and functional importance. Several surgical techniques have been used to treat such pathologies often with high morbidity and lacking the ability to address smaller contour defects. The minimally invasive technique of fat transplantation has evolved rapidly within the last few decades. The objective of this paper is to present the versatility and applicability of fat transplantation in a wide range of contour deformities in the craniofacial region. We share our experiences in treating 13 patients with autoimmune disorders, congenital malformations, and acquired defects. Future perspectives of fat transplantation in the field of craniofacial reconstruction are discussed.

## 1. Introduction

Gustav Neuber performed the first autologous fat transfer in 1893 for treatment of adhesive scars due to childhood tuberculous osteitis. He transferred fat parcels from the upper extremity to the infraorbital margin [1]. Since then refinements have improved the technique of fat transfer, primarily due to Coleman's systemization of fat transfer techniques in the 1990s, focusing on atraumatic fat transfer [2]. Initially autologous fat transplantation was considered an aesthetic procedure. However, autologous fat transfer is now considered a valid option in reconstructive surgery as well as in correction of scars [3–11]. The purpose of this study is to describe our experiences and the versatility of autologous fat tissue transfer in patients with a wide variety of facial disfigurations caused by trauma, inflammatory, infectious, or congenital conditions as well as after excision of tumors.

## 2. Materials and Methods

Medical records of 13 treated patients from August 2012 to July 2014 at the Department of Plastic Surgery at Aarhus University Hospital were reviewed. All patients who received fat transplantation to the head and neck area were included. Preoperative and postoperative standardized photographs were used to evaluate outcome at postoperative follow-up.

### 2.1. Surgical Technique

One patient was treated under sedation and local anesthesia, while all other procedures were performed under general anesthesia. Fat harvest was performed as described by Coleman [2]. A Sattlers or Khouris 3 mm extraction cannula connected to a 10 mL Luer-Lock syringe was used to harvest fat. Manual vacuum was created progressively to minimize destruction of adipocytes. Fat was harvested from the flanks, abdomen, breast, and thigh. The harvested fat was then centrifuged at 1800 rpm for 3 minutes and the supernatant oil and liposuction fluid were removed. In patients with scar tissue, rigottomy with 3-dimensional micromeshing was performed using a 12- to 18-gauge needle. The prepared adipose tissue was evenly distributed subcutaneously, fan-shaped, dropwise and evenly with a 21/20 G PIXL- or Coleman-cannula with a 3 mL syringe. Injection sites were closed with resorbable sutures and steristrips. Postoperative regimen was standardized with elevated headrest to 30 degrees and patients were instructed in avoiding pressure on the recipient site for 7 days. During the first 3 postoperative days the patients wore a protective nonadhesive dressing. Most of the patients at our department have a clinical follow-up 3 months postoperatively.

## 3. Results

The study population consisted of 13 patients as follows: 7 males and 6 females. The mean age was 32 years (range 9–62). No complications were observed in the 22 procedures performed. The mean number of series of fat transplantations, the patients had, was 1.69 (range 1–3). The mean volume of injected fat was 19.41 mL. (range 5–49.5). Ten patients had undergone previous surgical procedures, such as a Le Fort 1-osteotomy and a paramedian forehead flap. Mean follow-up was 10.6 months (range 1–22).  Table 1 summarizes the demographics and treatments of patients.

### 3.1. Autoimmune Disorders

#### 3.1.1. Scleroderma

**Figure 1:**
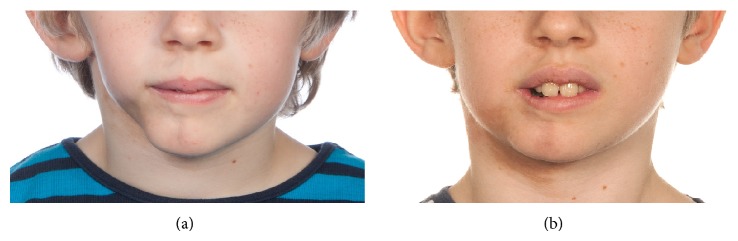
(a) Before fat transplantation. (b) Three months after third series of fat transplantation.

Scleroderma is an autoimmune systemic disorder of unknown etiology characterized by microvasculature damage and fibrotic changes of involved tissue. Scleroderma is classified as either diffuse with involvement of internal organs or limited when internal organs are not affected. Both limited and diffuse types can involve the skin of the face and may cause aesthetic disfiguration and disability in eating, drinking, and orthodontic care [4]. We treated a 9-year-old patient with localized scleroderma (Figures 1(a) and 1(b)). The skin of the chin and right side of the mandible were affected with atrophy and hyperpigmentation. The patient had undergone treatment with methotrexate. Due to slender stature, donor sites were limited and fat was therefore harvested from the gluteal and thigh regions. Three series of transplantations were done. Clinical controls have thus far shown good results with regard to volume retention. Unfortunately pigmentation of the skin has remained unchanged. However, the bony atrophy of the right side of the mandible has not progressed indicating a beneficial effect.

#### 3.1.2. Systemic Lupus Erythematosis

Lupus erythematosis is an autoimmune connective tissue disease with a variety of clinical presentations and can affect different organ systems. Women are more often affected than men, with peak presentation being from late teens to the 40s [12]. Facial involvement is often in the form of a malar rash (butterfly rash). A 62-year-old patient with systemic lupus erythematosus was treated for thinning of the subcutaneous tissue in the area of the classical malar rash and defects of the sternum and both breasts. The procedure was done under sedation supplemented with local anesthesia. Additional series of fat transplantations were planned in sedation with local anesthesia.

### 3.2. Acquired Defects

#### 3.2.1. Necrotizing Fasciitis

Necrotizing fasciitis is an uncommon condition characterized by infection of the fascia and may involve the subcutaneous tissue. It can rapidly progress to systemic toxicity and even death. All parts of the body can be involved. Management consists of immediate debridement and administration of antibiotics [13]. Autologous fat transplantation is a common treatment for scar correction, yet very little literature exists on the use of fat transplantations in the correction of scars after necrotizing fasciitis. We used the technique in a patient with necrotizing fasciitis of the epiglottis and involvement of the anterior part of the neck a year after infection. Following primary surgical intervention the patient had extension deficit of the neck, because of adherence of the scars to underlying tissue. After only two series of fat transplantation with rigottomy of adherent scars and a V-Y advancement flap from the left side of the neck, the patient experienced increased symmetry and mobility of the neck. Further treatment was therefore unnecessary.

#### 3.2.2. Abscess

A 36-year-old patient was treated for a contour deformity of her left cheek, which she sustained more than three decades earlier following drainage of an abscess of the cheek. The first series of treatment had modest effect and further treatments were scheduled.

#### 3.2.3. Gunshot

A study by Arcuri et al. investigated 19 patients who underwent posttraumatic reconstruction of maxillofacial deformities with fat transplantation. They achieved excellent results with adequate facial balance after clinical and software analysis [6]. We treated a 44-year-old patient, who had suffered a gunshot wound to the face two decades ago, resulting in enucleation of the right eye and the need for reconstruction of the right lower eyelid with a paramedian forehead flap. The patient presented with lack of filling in the right zygomatic region. 5 mL autologous fat was injected. There was no follow-up on this patient.

### 3.3. Congenital Malformations

#### 3.3.1. Hemangioma

Hemangiomas are congenital vascular tumors of rapidly dividing endothelial cells. They frequently occur as solitary lesions of the head and neck. Hemangiomas are considered the most common tumor in infancy, the majority of which regress spontaneously. Esthetic sequelae however frequently persist, leaving fibro-fatty residual scars. Treatment options include surgery, laser, nonselective beta-blockers, systemic or intralesional corticosteroids, chemotherapy, or combinations [14]. We treated a 16-year-old patient with sequelae after regression of a hemangioma affecting the right half of the face. The patient had undergone CO_2_ laser treatment with modest effects on scar development. Fat transplantation was performed without any excessive bleeding or hematoma. Clinical follow-up of 6.5 months has been without recurrence of the hemangioma. Softening of the skin was achieved after the first series of fat transplantation. Further treatments were planned to restore the natural contours of the face.

#### 3.3.2. Mesenchymal Chondrosarcoma

Mesenchymal chondrosarcoma is a very rare tumor accounting for 1% of sarcomas, with potential of highly aggressive behavior. Peak incidence is in the second decade of life, most commonly affecting the axial skeleton [15]. We performed three series of fat transplantations in an 11-year-old patient 9 months after surgical removal of a chondrosarcoma involving the right orbit, frontal, and temporal regions. Removal of the chondrosarcoma with osseous involvement resulted in enucleation of the right eye and resection of the right temporal muscle with contour defect and scar formation. Furthermore the patient underwent proton radiation of the involved area and chemotherapy prior to fat transplantation. After three fat transplantations the patient's facial contour defects were corrected to such a degree that fitting of a prosthetic right eye was possible. After 15 months of clinical follow-up there was no sign of recurrence.

#### 3.3.3. Treacher Collins Syndrome

Treacher Collins syndrome (mandibulofacial dysostosis) is a rare autosomal dominant congenital disorder. Characteristic abnormalities include hypoplasia of the facial bones, particularly the maxilla, mandible, and zygoma. Teeth may be widely spaced, with a high palate often with cleft. Abnormal position and shape of the auricle are common, often accompanied by conductive hearing loss as well as ophthalmic abnormalities such as downward slanting of palpebral fissures. Mental retardation and psychomotor delay may also occur [16]. There are several studies on fat transplantation in patients with Treacher Collins syndrome [3, 7]. Guibert et al. performed a three-dimensional evaluation of transplanted children for objectively quantifying graft survival. The study found a 40% survival rate of the graft in line with prior MRI studies [3]. Lim et al. found that there was a 7.67% increase in symmetry in patients treated with fat transplantations [7]. Patients in these studies were typically treated in adolescence. We treated a 57-year-old patient who had undergone multiple reconstructive operations due to Treacher Collins syndrome, including a left side Tessier 7 facial cleft. The patient had pronounced adherence to the underlying tissue at the scar after the cleft repair. The patient wished a reduction of breast volume and two series of fat transplantations were planned using donor fat transplanted from the breasts. Good clinical results were achieved after the first transplantation and the engraftment of fat was clinically evident; still further corrections were necessary.

#### 3.3.4. Hemifacial Microsomia

**Figure 2:**
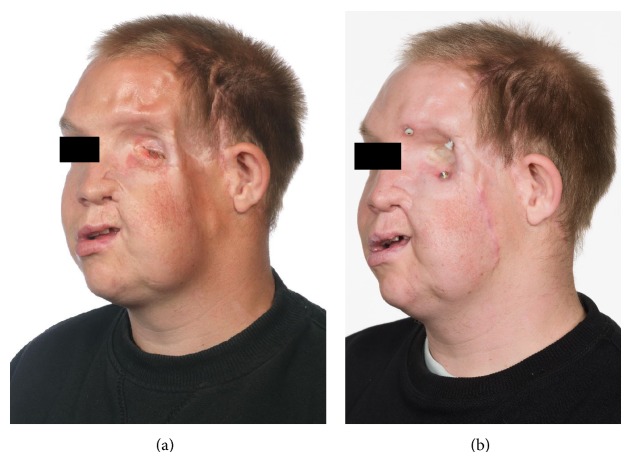
(a) Before fat transplantation. (b) Three months after two series of fat transplantation. The increased volume surrounding the orbita allowed better adaptation of the patient's prosthetic eye.

Hemifacial microsomia is the second most common facial birth defect after clefts and is the result of dysmorphogenesis and hypoplasia of the first and second branchial arch. There is great phenotypical variation but in particular mandibular, maxillary, and zygomatic hypoplasia is seen. Ear malformations, abnormal tooth development, and abnormal orbita size and position as well as facial nerve involvement may be present in a varying degree [17]. We treated four patients with hemifacial microsomia, aged 19, 20, 31, and 41 years. All had undergone extensive reconstructive surgery. The primary goal of treatment for the youngest two patients was to increase facial symmetry and soften facial clefts. Softening of the scars was achieved after the initial treatments, whereas volume retention and facial symmetry were more demanding, requiring several series of treatment. The older patients had prostheses, one orbital (Figures 2(a) and 2(b)) and the other auricular. These patients had fat transplantations with good result to contour defects in proximity of the prostheses, thus minimizing focus on the prostheses.

#### 3.3.5. Neurofibromatosis Recklinghausen

Neurofibromatosis is an autosomal dominant disorder in which patients have a high risk of tumor development. Neurofibromas manifest as benign focal cutaneous, subcutaneous, or plexiform lesions derived from peripheral nerve sheaths, nerves, and nerve roots [18]. Craniofacial neurofibromas are very stigmatizing and are most often located at the orbital-temporal region [19].

**Figure 3:**
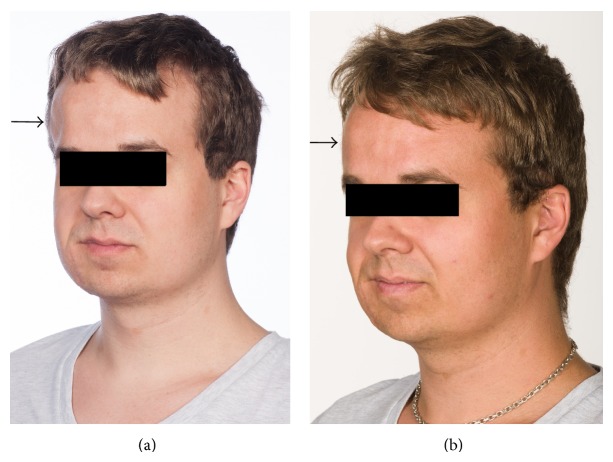
(a) Before fat transplantation contour defect on the right frontal region in the location of the black arrow. (b) Three months after one fat transplantation with a decreased contour defect on the right frontal region of the head in the location of the black arrow.

We treated a 21-year-old patient with neurofibromatosis Recklinghausen with autologous fat transplantation into a cranial contour deformity of the right temporal region (Figures 3(a) and 3(b)). The elasticity of the overlying skin allowed the transplantation of a large amount, 31 mL of fat, which had a lasting effect; further treatments were therefore not indicated.

## 4. Discussion

It is well accepted that survival of fat grafts depends on the fat drops having a maximum radius of 2 mm, so the transplant can survive by plasmatic imbibition until revascularization establishes a recipient capillary network. In addition, it is theorized that an increase in volume to a recipient site decreases the compliance of the tissue. This decrease in compliance results in an increase in interstitial fluid pressure which in turn decreases capillary circulation and subsequently graft survival [20]. It is consequently imperative that one considers every transplantation unique as a delicate balance between graft volume and recipient site.

Autologous fat transplantation shows its elegance when detailed modulation is needed for small contour defects that attract the eye. The primary challenge when conducting procedures with need for such high detail is the large variation in graft take, ranging from 25 to 80% [21]. Fortunately a recent randomized control study has shown that adipose-derived stem cells have a much larger and more consistent graft take. Thus, this allows surgeons to perform fewer procedures with less overcompensation and increasing the level of detail [21]. There is, though, an ongoing debate on the use of adipose-derived stem cells in cancer patients. It has been hypothesized that adipose-derived stem cells could reactivate or increase activity of malignant cells [22]. Thus far several large studies have shown that autologous fat transplantation does not increase the risk of breast cancer recurrence in patients [23].

In our experience, fat transplantation into radiated areas requires multiple treatments due to increased adherence to underlying structures and is hence clinically demanding. It is therefore of clinical interest that Rigotti et al. found promising results using adipose-derived stem cells to treat radiation induced skin lesions [24].

We believe that the role of fat transplantation in the correction of craniofacial deformities* currently* lies after major reconstructive surgery. Autologous fat transplantation is ideal in settings which require modulations of contour defects, which cannot be addressed during primary surgical intervention. Regarding the future of craniofacial reconstruction, we are convinced that fat transplantation, possibly with the use of stem cells, will play an increasingly important role. In the future and with the rapid ongoing evolution of fat transplantation, the need for larger reconstructive surgical procedures will diminish. This will undoubtedly reduce the associated morbidity and donor site morbidity. Hopefully in the near future a safe and less invasive approach to craniofacial reconstruction will allow reconstruction of patients earlier in life, limiting the psychological impact of aforementioned deformities. Continued development in the field of fat transplantation requires further research in fat and stem cell transplantation, including documentation of clinical experience.

## 5. Conclusion

The present report has highlighted the multiple utilities of fat transplantation in patients with various deformities of the craniofacial region. In our experience, autologous fat transplantation to the face, head, and neck with rigottomy, when deemed necessary, is a safe procedure, and we observed no complications in 13 patients who in total had 22 procedures. Improvement of contours and softening of the skin were achieved. Fat transplantation has a wide range of application in contour deficits, scar adherence and disfiguration caused by trauma, inflammatory, infectious, or congenital conditions and after tumor removals. Although multiple procedures are usually required, the procedures are minimally invasive and hospitalization is short. We also found that sedation and local anesthesia can be sufficient when transplanting fat into the head and neck area, as has been described in prior studies [6, 25, 26].

We hope that our experiences will help other surgeons when contemplation surgical techniques in similar clinical cases. Hopefully this report will also contribute to the design of further studies in which the survival of transplanted fat into craniofacial deformities can be verified by, for example, MRI. We also believe that patient's satisfaction should be registered in such studies as we found that fat transplantation in addition to volume formation may influence softness and compliancy of the surrounding skin.

## Figures and Tables

**Table 1:** Demographics and treatments of patients.

Age	Sex	Diagnosis	Number of procedures	Donor site Abdomen (A) Thigh (T) Breast (B)	Months since initial surgery	Further transplantations needed	Transplanted volume (mL)
19	Female	Hemifacial microsomia	3	T, T, T	12	Yes	19, 23, 14.5
57	Male	Treacher Collins	1	B	8	Yes	17
49	Female	Necrotizing fasciitis	2	A, A	13	No	20, 19
44	Male	Gunshot	1	A	7	To be decided	5
20	Male	Hemifacial microsomia	1	A	1	Yes	13
16	Female	Hemangioma	1	A	6.5	Yes	15.5
11	Female	Mesenchymal chondrosarcoma	3	A, A, A	15	To be decided	13, 15, 17.5
21	Male	Neurofibroma (Figures 3(a) and 3(b))	1	A	16	No	31
62	Female	Systemic lupus erythematosis	1	A	4	To be decided	8.2
9	Male	Scleroderma (Figures 1(a) and 1(b))	3	T, T, T	22	To be decided	14, 22, 36
36	Female	Abscess	1	T	7	Yes	12
31	Male	Hemifacial microsomia (Figures 2(a) and 2(b))	2	A, A	17	Yes	12, 12
41	Male	Hemifacial microsomia	2	A, A	9.5	To be decided	39, 49.5
